# Quantification of Desiccated Extracellular Vesicles by Quartz Crystal Microbalance

**DOI:** 10.3390/bios12060371

**Published:** 2022-05-27

**Authors:** Vasiliy S. Chernyshev, Mikhail Skliar

**Affiliations:** 1 Center for Photonic Science and Engineering, Skolkovo Institute of Science and Technology, Bolshoy Boulevard 30, bld. 1, 121205 Moscow, Russia; 2 School of Biological and Medical Physics, Moscow Institute of Physics and Technology, Institutsky per. 9/7, Dolgoprudny, 141700 Moscow, Russia; 3 The Nano Institute of Utah, University of Utah, 36 S. Wasatch Dr, Salt Lake City, UT 84112, USA; misha@chemeng.utah.edu; 4 Department of Chemical Engineering, University of Utah, 50 S. Central Campus Dr, Salt Lake City, UT 84112, USA

**Keywords:** extracellular vesicles, quartz crystal microbalance, biosensor

## Abstract

Extracellular vesicle (EV) quantification is a procedure through which the biomedical potential of EVs can be used and their biological function can be understood. The number of EVs isolated from cell culture media depends on the cell status and is especially important in studies on cell-to-cell signaling, disease modeling, drug development, etc. Currently, the methods that can be used to quantify isolated EVs are sparse, and each have limitations. In this report, we introduce the application of a quartz crystal microbalance (QCM) as a biosensor for quantifying EVs in a small drop of volatile solvent after it evaporates and leaves desiccated EVs on the surface of the quartz crystal. The shifts in the crystal’s resonant frequency were found to obey Sauerbrey’s relation for EV quantities up to 6 × 10^7^, and it was determined that the biosensors could resolve samples that differ by at least 2.7 × 10^5^ EVs. A ring-shaped pattern enriched in EVs after the samples had dried on the quartz crystal is also reported and discussed. QCM technology is highly sensitive and only requires small sample volumes and is significantly less costly compared with the approaches that are currently used for EV quantification.

## 1. Introduction

Extracellular vesicles are membrane-bound particles that contain proteins, nucleic acids, and other cargo inherited from the mother cell that secretes them into the extracellular space [1,2,3,4]. Such vesicles have been found to be very stable and are released into circulation by nearly all cell types for short- and long-range intercellular signaling, with their molecular cargo being delivered via fusion with the recipient cells [5,6]. The stability of these vesicles in biological fluids such as blood, urine, saliva, and breast milk allows for EV characteristics such as the concentration, size, or presence of specific biomarkers to be determined, and this information can potentially be used for medical screening and diagnostics [3]. For instance, the rate of EV secretion by cells and the EV concentration have already been shown to depend on health status and environmental conditions [3,7].

In samples, EV quantification is currently limited to only a few techniques [8]. Nanoparticle tracking analysis (NTA) is based on the light scattered by particles and allows for nanoparticles to be quantified in the field-of-view volume. It is currently one of the most commonly used techniques for EV characterization. However, such instrumentation has drawbacks, such as its high cost, the need to optimize the dilution of the EV sample to obtain approximately 30–100 particles in the field-of-view, and the need to perform multiple technical repeats to account for variability within the performed measurements. Tunable resistive pulse sensing (TRPS) is another characterization method which is based on the Coulter principle and that allows for single particles that pass through a nanopore to be counted [9]. The resistance increases when the particles pass through, generating a pulse which is proportional to the particle volume and allowing EV enumeration to be performed. Flow cytometry based on the optical detection of particles emitting fluorescent or scattered light is another quantification technique that allows for the EVs passing through the interrogation point at a known flow rate to be counted [10]. This method relies on fluorescent-based immunostaining to provide an individual count of the EVs containing specific markers. Enzyme-linked immunosorbent assay (ELISA) is another method for EV quantification which is based on an abundance of labeled biomarkers such as membrane proteins. However, similar to flow cytometry analysis, ELISA only allows for EVs containing a specific biomarker to be quantified, and the amount can vary significantly depending on the EV source [8].

A quartz crystal microbalance (QCM) is a piezoelectric sensor that allows the mass to be measured at the nanogram scale by monitoring changes in the frequency of a resonating quartz crystal caused by the adsorbed mass (e.g., molecules or nanoparticles) [11,12]. This method includes a broad range of applications such as gas phase detection [13], immunosensors [14], DNA biosensors [15], and deposition [11,16]. The biomimetic coatings achieved by molecular imprinting strategies highlight the ability to achieve a high sensitivity, selectivity, and rapid response time and simulate natural receptors [17]. QCMs have previously been shown to be superior mass detectors for liquid chromatography, because they compare the resonant frequency of the oscillating crystal before liquid sample deposition and after solvent evaporation, with the remaining residue causing frequency changes [18]. A similar approach was later presented and shown to be suitable for determining the concentrations of *Si* and *Ag* nanoparticles in a sample [19].

Recently, QCMs were shown to be applicable for the label-free characterization of EVs, including their quantification and interaction kinetics via functionalizing the surface of the electrode with specific antibodies and capturing EV subpopulations [20,21,22,23]. Other reports have shown the ability of a monolayer of cells to form on the gold electrodes and the ability to monitor the release of microvesicles using a biosensor based on a QCM [24]. However, QCMs have not been utilized to analyze and quantify all of the EVs secreted by cultured cells, which is important when performing signaling studies or when developing tissue and disease models. We present a novel high-precision approach for the quantification of isolated EVs after sample desiccation on the crystal surface of a QCM sensor. The EVs extracted from cell culture supernatant using a precipitation technique were resuspended in an ammonium acetate solvent which is commonly used as a volatile buffer for liquid chromatography–mass spectrometry. This enabled the solvent of the EV sample to evaporate within a short period of time, leaving the desiccated EVs on the quartz surface. First, we performed biosensor calibration using the EVs obtained from different cell lines of varying concentrations that had been determined by NTA. The quartz crystal frequencies were measured before an EV sample with a chosen volume was deposited on the crystal, and after solvent evaporation. The number of EVs was linearly correlated with the frequency changes determined by the QCM. To test the calibration curve, we used additional samples with different EV concentrations. We show that this QCM-based biosensing approach allows for the number of EVs present in a sample to be determined, even when the volume is only a few microliters, in a short period of time and with high precision. In addition, QCM biosensors are inexpensive compared with current, commonly used approaches for EV quantification.

## 2. Materials and Methods

### 2.1. Materials 

Bovine serum albumin (BSA) was purchased from Sigma Aldrich (Burlington, MA, USA). For dot blotting, an antibody array (Exo-Check #EXORAY200A-4) was purchased from SBI. The developer solution used for dot blotting (SuperSignal West Femto Chemiluminescent Substrate, #34094) was purchased from ThermoFisher Scientific (Waltham, MA, USA). Ammonium acetate (AA) salt (#A1542) was purchased from Sigma-Aldrich. Cell culture media RPMI-1650 (#11875093), DMEM (#A4192101), DMEM-12 (#11320033), and F-12K (#21127030) were purchased from ThermoFisher Scientific. Protein LoBind tubes were purchased from Eppendorf (Hamburg, Germany).

### 2.2. Cell Culture

Prior to cell culture, MDA-MB-231, MCF7 human breast cancer cells, MCF10A benign human epithelial breast cells, PC3, LNCaP, and 22Rv1 human prostate cancer cells were stored in liquid nitrogen. Cell culture medium RPMI-1650 was used for the LNCap and 22RV1 cell cultures. F-12K medium was used for the PC3 cell culture. DMEM was used for MCF7 and MDA-MB-231 cell cultures. DMEM-F12 was used for the MCF10A cell culture. The cell line was thawed, plated on 150 mm plates, and aerated by 95% air and 5% CO_2_ at 37 °C. Once the cells settled down, the media were changed (approximately 24 h after plating). The plate was then split at a 1:10 ratio, and 10 plates were cultured. Each plate contained 20 mL of media. To isolate the EVs, flasks were brought to a confluence of 90–100%. The culture medium was then replaced with a culture medium that did not contain fetal bovine serum 48 h before it was collected for EV isolation. After 48 h, the media from these plates were harvested and pooled. The media were then split into 30 mL tubes, and these tubes were centrifuged at 300× *g* for 8 min to remove any dead cells that could have been present in the medium followed by further centrifugation at 3000× *g* for 15 min to remove any other cell debris. Each supernatant was then transferred to a new sterile 50 mL tube and kept at −20 °C prior to EV isolation.

### 2.3. EV Isolation by ExoQuick-TC

The EVs were isolated with a commercially available ExoQuick-TC kit based on precipitation, a method that was previously used and reported in other studies [25,26,27]. In short, 6 mL of ExoQuick-TC precipitating solution was added to each 30 mL cell culture supernatant (1:5 volume ratio), well mixed, and kept at 4 °C overnight. The mixture was then centrifuged at 1500´ *g* for 30 min at room temperature. After centrifugation, the supernatant was discarded and the EVs appeared as beige pellets. The remaining EV pellets were spun for another 5 min at 1500× *g* to separate the residual ExoQuick-TC solution, which was removed without disturbing the pellets. The obtained pellets were resuspended in PBS or 2 mM AA. The obtained EV samples were then separated into 100 µL aliquots and stored in LoBind Eppendorf tubes at −20 °C until use.

### 2.4. Dot-Blotting

To assess the quality of EV isolation achieved using Exoquick-TC, the expression of the MCF7 EV surface biomarkers was evaluated by the dot blot antibody array (Exo-Check). A 150 µL volume of EVs was lysed by adding 600 µL of the lysis buffer and vortexing the mixture for 15 s. Subsequently, we added 9.4 mL of the binding buffer that was supplied with the array. The array was wetted in 5 mL of distilled water for 2 min at room temperature. After decanting the water from the membrane, 10 mL of the EV–lysate–binding mixture was pipetted onto the Exo-Check membrane and incubated for 12 h on a shaker at 4 °C. The mixture was then discarded, and 10 mL of wash buffer was added and gently agitated on the membrane surface for 5 min at room temperature. After removing the wash buffer, 10 mL of detection buffer was pipetted on the membrane surface and incubated for 2 h on a rocker at room temperature. The detection buffer was then discarded, the surface was washed three times with the wash buffer, and 2 mL of the developer solution (SuperSignal West Femto Chemiluminescent Substrate) was added so that it completely covered the array membrane. After 2 min, the developer solution was discarded, and the array’s response was read using the Bio-Rad ChemiDoc XRS Imager System (Hercules, CA, USA). The obtained grayscale image was parsed into twelve areas corresponding to two positive controls (a bright signal indicates that the detection reagents are working properly), a blank spot (establishes a background signal), a negative control to characterize cellular contamination (cis–Golgi matrix protein, GM130), and eight surface proteins (ANXA5, TSG101, FLOT1, ICAM, CD63, CD81, ALIX, and EpCAM) that are known to be expressed to various degrees on the surfaces of different EV types.

### 2.5. Nanoparticle Tracking Analysis (NTA)

The EV concentration and hydrodynamic size distribution were determined using the Nanosight instrument (model LM10; Malvern Panalytical, Salisbury, UK) by illuminating the sample with a 40 mW violet laser (405 nm wavelength), the light being scattered by the EVs was captured with a high-sensitivity sCMOS camera (OrcaFlash2.8, Hamamatsu C11440), and the software provided by the manufacturer (Nanosight Version 3.0, Malvern Panalytical, Salisbury, UK) was used to analyze the results. The minimal track length was set to automatic, the blur size was set to 1 pass, the gain was set to 1, the brightness was set to 12, and the detection threshold was set to 5. The viscosity of 2 mM of AA was assumed to be equal to the viscosity of water, which depended on temperature, and was adjusted automatically based on the temperature measurements. The temperature of the cells was measured manually and remained at 20 °C, with a maximum fluctuation of 0.1 °C being observed throughout the nanoparticle tracking process. The viscosity of water at these temperatures is nearly constant and equal to 1 cP. Prior to analysis, the EV samples were diluted 1:100–1:2000 in PBS to obtain approximately 30–100 particles in the field-of-view, which translates to a concentration of approximately 0.1–2 × 10^9^ particles/mL for each measurement. Five 60 s videos, each consisting of 1400 frames, were recorded and analyzed using NTA software with the described settings. The NTA software reported the EV concentration, size distribution, its mode, mean, and the standard deviation.

### 2.6. Quartz Crystal Microbalance (QCM)

A titanium/gold electrode with a polished quartz crystal that was 1 inch in diameter was used as the biosensor crystal in this study. A 5 MHz QCM200 (Stanford Research Systems, Inc, Sunnyvale, CA, USA) quartz crystal microbalance instrument was used to perform the mass measurements. The RS-232 port was connected to a desktop computer (HP Z400 Work-station), and the LabVIEW stand-alone application was used for data acquisition. The compensation switch of the QCM200 was set to hold, and the ten-turn dial was set to 8.0 (dry operation setting). Prior to introducing a sample on the crystal center, the oscillation frequency was given 15 min to equilibrate. A 5 µL volume sample was then manually deposited to the center of the biosensor crystal in the form of a sessile drop using a 10 µL pipette. The solvent (2 mM AA) in which the EV samples were prepared was allowed to evaporate while the resonant frequency was recording. Measurements were stopped 15 min after the complete evaporation of the solvent and the QCM frequency of the oscillation reaching an equilibrium value. To obtain a calibration curve, EVs that had been isolated from the cell culture media of five different cell lines with different concentrations were used to account for the potential differences in the biophysical properties of the EVs produced by different cells. Images of the crystal with a desiccated sample were obtained after the completion of the QCM measurements and after the complete evaporation of the solvent using an optical microscope containing a digital camera with a 1024 × 768-pixel resolution (Imaging Source, Charlotte, NC, USA) and MATLAB software (MathWorks, Natick, MA, USA). Each sample was measured at least three times at room temperature and under atmospheric pressure.

### 2.7. Transmission Electron Microscopy (TEM)

In the MCF7 EVs, TEM imaging was used to confirm EV isolation by visualization. Negatively stained specimens were prepared according to a standard method. An EV sample (~3.5 µL) was placed on formvar–carbon-coated copper mesh TEM grids, which were glow-discharged to impart hydrophilicity. The sample was allowed to adhere to the grid for approximately 1–2 min before the liquid was blotted, and the surface washed with deionized water when necessary. A small droplet (~3.5 µL) of 1% uranyl acetate solution was then placed on the grid and blotted away after ~20 s. All of the steps were performed at room temperature. The dried specimens were imaged at 120 kV (TEM JEM1400Plus, JEOL USA, Peabody, MA, USA), and the images were recorded on a Gatan Orius camera (Gatan, Inc., Pleasanton, CA, USA).

### 2.8. Scanning Electron Microscopy (SEM)

Prior to SEM imaging, the MCF7 EV sample was diluted at a ratio of 1:10 in 2mM AA. A glass slide was gently cleaned with nitrogen gas and placed on the specimen stage of the SEM (FEI NanoNova 630 High Resolution SEM). Samples totaling five microliters in volume were then deposited on the glass slide and allowed to dry at room temperature and atmospheric pressure. The sample was then imaged at 0.98 Torr using a low-vacuum secondary electron detector at magnifications in the 15,000–55,000× range.

### 2.9. Atomic Force Microscopy (AFM)

The hydrated MCF7 EVs isolated by ExoQuick-TC were electrostatically immobilized on the mica surface with a 10 mM concentration of a surface charge-modified NiCl_2_ solution and were characterized by atomic force microscopy (AFM) according to the previously reported protocol [28]. AFM imaging was performed using a Veeco Multimode Nanoscope V controller (Veeco Instruments Inc., Town of Oyster Bay, NY, USA). We pipetted ~40 µL of PBS on the mica surface, where the EVs were adsorbed and scanned over a 5 × 5 µm^2^ area in 512 lines with an MLCT triangular cantilever (175 µm nominal length, 22 µm width, 0.07 N/m spring constant, and 20 nm tip radius; Bruker, Billerica, MA, USA) held in a Bruker’s MTFML probe holder. The scan rate in the tapping mode was set to 0.8 Hz, and the drive frequency was maintained at ~7 kHz to obtain height images.

## 3. Results

### 3.1. EV Isolation

The goal of this study was to quantify the EVs that were initially present in the 2 mM ammonium acetate (AA) solvent by measuring the mass before the sample was introduced to the quartz crystal of the QCM and after sample desiccation. The EVs that were used in this study were isolated from MCF7, MCF10a, 22Rv1, LnCaP, MDA-MB-231, and PC3 cell culture media using ExoQuick-TC. The mode and mean hydrodynamic size ranged from 113 to 135 nm and from 147 to 220 nm, respectively (Table 1).

**Figure 1:**
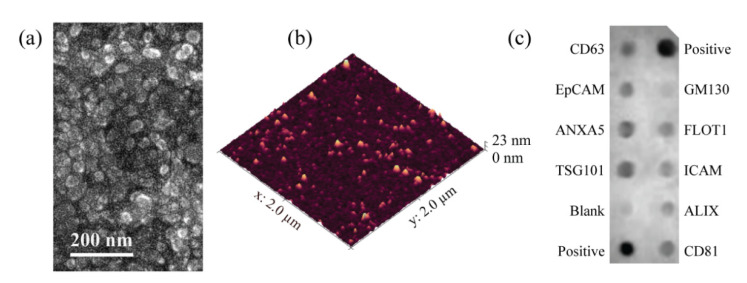
Characterization results of the EVs isolated from cell culture media by ExoQuick-TC. (**a**) EM image of EVs isolated from the MCF7 cell culture medium. The particles in the EV sample were within the 20–60 nm range. (**b**) AFM scan of hydrated EVs isolated from the MCF7 cell culture medium and immobilized on a mica substrate modified with NiCl_2_. (**c**) Dot blotting results of EVs isolated from the MCF7 cell culture medium showing the expression of CD63, EpCAM, ANXA5, TSG101, FLOT1, ICAM, ALIX, and CD81 that are characteristic of EVs and the negative expression of GM130, confirming sample purity.

TEM showed the presence of rounded desiccated vesicles with a geometric size < 100 nm (Figure 1a). The representative AFM tapping-mode height image of the surface-bound EVs maintained in PBS is shown in Figure 1b. The AFM images indicated shape distortion from the expected globular geometry [29,30,31,32] caused by the electrostatic attraction of the EVs, which are known to have a negative zeta potential, to the mica surface, which was modified to be positively charged. The peak height of the EVs protruding above the surface was in the range of 5–20 nm, and X–Y diameter ranged from 70 to 120 nm. Such values agree with previous reports on EVs of different origins [28,33,34]. Dot blotting showed the expression of the CD63, EpCAM ANXA5, TSG101, FLOT1, ICAM, ALIX, and CD81 markers and the absence of contaminant GM130 in the MCF7-derived EV sample that was used to validate the ExoQuick-TC isolation protocol (Figure 1c).

### 3.2. QCM Biosensor Calibration

**Figure 2:**
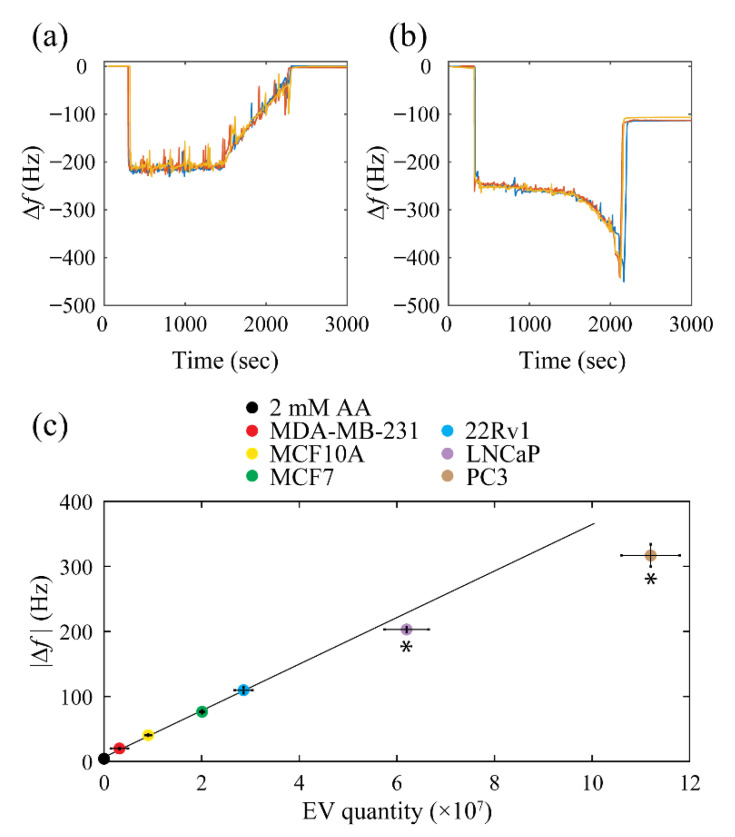
Results obtained by the QCM biosensor after the analysis of the EVs that were resuspended in 2 mM AA to construct a calibration curve. Examples of frequency changes after the introduction of (**a**) 2 mM AA without EVs and (**b**) 2 mM AA containing EVs isolated from the 22Rv1 cell culture medium. (**c**) Calibration curve produced by performing measurements (three repeats) of EVs isolated from different cell lines with a known concentration obtained by NTA. The asterisks signify data points deviating from the Sauerbrey relation, which were excluded from the linear regression, to find the relationship between the EV concentration and the differences in frequency before sample introduction and after desiccation. Error bars show the standard deviation of EV quantity determined by NTA and the standard deviation of the frequency shift obtained by the QCM biosensor. The R^2^ of the linear fit was determined to be 0.996.

A 5 µL volume drop was manually deposited on the center of the biosensor crystal to form a sessile drop with a diameter that ranged from 2.4 to 2.8 mm. Figure 2a,b show the frequency changes that were frequently observed after the introduction of the sessile drop of 2 mM AA solvent in the absence or presence of isolated EVs, respectively. The frequency dropped sharply after the liquid was deposited, and during the initial evaporation (<20 min), no significant changes in frequency were observed. However, after a longer time, there was a distinct drop in the frequency (200–250 Hz) (Figure 2b). After the complete evaporation of the solvent, the frequency increased sharply and reached a steady state. The time needed for sample desiccation and for the frequency to reach a steady state appeared to be consistent between repeats, with the variations in the frequency at equilibrium not exceeding 5%.

The QCM biosensor was calibrated using the EVs isolated from six different cancer cell lines to find the applicability range of the Sauerbrey relation:

(1) $$∆f=-C_{f}∆m$$

where ∆*f* is the frequency change (Hz), ∆*m* is the change in mass per unit area (µg/cm^2^), and $C_{f}$ is the crystal sensitivity factor (56.6 Hz µg^−1^ cm^2^). Analyzing the EVs obtained from different cell lines accounts allows for potential variability in their biophysical properties. The EV concentration chosen for the QCM calibration curve ranged from 6.3 × 10^8^ to 2.2 × 10^10^ particles per mL. EV samples with a known volume and EV concentration were deposited, and the frequency was recorded. AA solvent (2 mM) without EVs present was used as a control to account for the effect of any leftover residue after its evaporation on the frequency changes. After each measurement, the desiccated sample was also imaged with an optical microscope. After evaporation, the residue of the solvent not containing any EVs caused the frequency to remain 4.2 ± 0.5 Hz below the reference value that had been recorded before sample deposition. Taking the contribution of AA on the change in the total mass into account, the frequency change can be expressed as:

(2) $$∆f=∆f_{\mathrm{EV}}+∆f_{\mathrm{AA}}$$

where $∆f_{\mathrm{EV}}$ and $∆f_{\mathrm{AA}}\;$ represent the effects of EVs and AA on the crystal’s frequency changes, respectively. The Sauerbrey relation can be rewritten in a form that relates the EV quantity to the frequency shifts caused by EV deposition in the following form:

(3) $$N_{\mathrm{EV}}=-c_{\mathrm{EV}}\Delta f_{\mathrm{EV}}$$

where $N_{\mathrm{EV}}$ is the number of EVs present in the sample, and $c_{\mathrm{EV}}$ is a constant term that incorporates the EV mass, the area of the desiccated sample, and the crystal sensitivity factor. Regression indicated the best linearity when the quantity of the desiccated EVs was less than 6 × 10^7^ (Figure 2c), allowing the relationship between the resonance frequency and the number of EVs present on the biosensor to be represented in the following form:

(4) $$N_{\mathrm{EV}}=-2.708\times 10^{5}\left(∆f-∆f_{\mathrm{AA}}\right).$$

Considering the impact of the AA residue after desiccation and the variability between repeats, the lower limit of the biosensor was found to be 3.0 × 10^5^ EVs. The calibration results indicate that the biosensor is practical for analyzing desiccated EVs, with their quantity ranging from 0.03 to 6 × 10^7^, and it can differentiate between samples that differ by a minimum of 2.7 × 10^5^ EVs. The time required to analyze a 5 μL sample was found to be 30 min on average.

### 3.3. Testing the Calibration Curve

To evaluate the precision of the calibration curve, additional EV samples were tested by varying the EV concentration to be within the 0.03–2.5 × 10^7^ range and analysing the calibration curve after performing the QCM measurements at least three times. The results were compared with the values that were obtained by NTA (Table 2). The results show that the calibration curve is applicable for quantifying the desiccated EVs that were resuspended in the AA solvent in the identified range, with the standard deviation not exceeding 10%.

### 3.4. EV Desiccation

**Figure 3:**
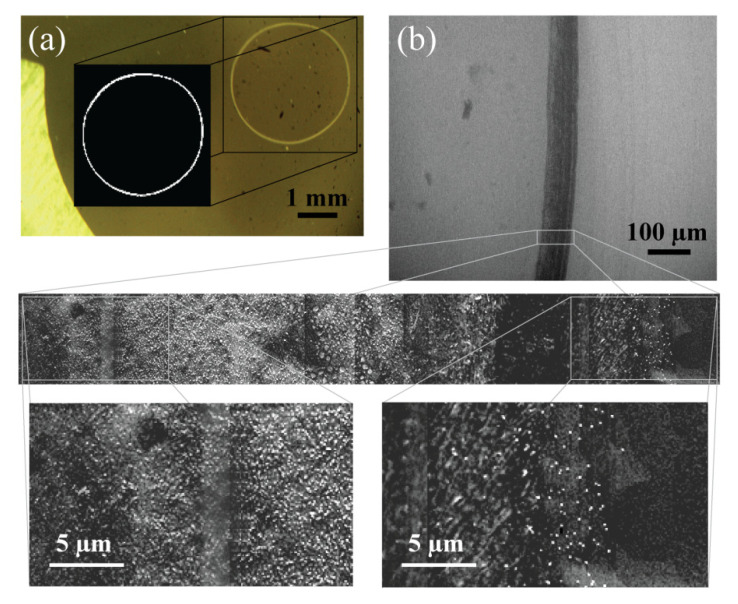
Analysis of the ring-shaped pattern after EV sample desiccation. (**a**) Image of a desiccated EV sample on the surface of the quartz crystal showing a ring-shaped stain and the measurement of its area performed using custom MATLAB software. (**b**) The edge of a dry sessile drop with a clearly defined boundary analyzed by SEM. The magnified image shows a slice of the ring and the presence of accumulated EVs.

After each QCM measurement, the desiccated sample was imaged using an optical microscope. When the sessile drop of the solvent containing the EVs dried on the biosensor surface, a deposit appeared as a ring-shaped stain (Figure 3a).

To determine the location of the EVs and how they were distributed after the complete evaporation of the AA solvent, the procedure was repeated using a glass slide on which a 5 μL sessile drop was placed and allowed to dry at room temperature. SEM analysis was performed from the left to the right edge of the dry sample. It was found that the EVs were densely accumulated at the initial air–liquid–solid interface of the sample, leaving a stain with a thickness < 100 μm (Figure 3b). AA crystals were also observed (data not shown) but were distributed throughout the inspected area of the desiccated drop. Compared with the edge of the desiccated sample, no EVs were observed at the center. The images that were obtained after completing the QCM measurements were processed using a custom code written in MATLAB software to find the area of the observed ring-shaped stain where the EVs appeared to be concentrated. The mean area of the ring-shaped pattern ranged from 0.15 to 0.29 mm^2^ (Table 3). Hence, the mass of the desiccated sample can be estimated by combining the area of the agglomerated EVs and the Sauerbrey equation:

(5) $$M=-\frac{∆f_{\mathrm{EV}}A}{C_{f}}$$

where *M* is the total mass of the dry sample (µg) and A is the surface area of the ring-shaped residue enriched in EVs. The known total number of EVs that accumulated at the ring of the dry sample allows for the average mass of the EVs in each cell line to be estimated, and it was found to range from 0.13 to 0.18 fg (Table 3). Considering that EVs have a spherical shape [29,30,31,32] and a 1.10 g/mL density [35,36], the mean diameter of the EVs was found to range from 48 to 54 nm, which is in agreement with the sizes determined according TEM and SEM (Figure 1a and Figure 3b) and according to previous studies [29,37].

## 4. Discussion

A novel method for the quantification of the desiccated EVs in a sample using a QCM biosensor is reported in this study. QCM sensors take advantage of piezoelectric crystals and were first introduced by the Curie brothers at the end of the 19th century [38]. An expression associating the detectable shift in the crystal resonance frequency (∆*f*) with the deposited mass (∆*m*) was later presented by Günter Sauerbrey [12]. QCM technology has already been implemented in multiple fields due to its high sensitivity, low cost, and portability [39]. The spectrum of QCM applications includes biosensing, which has already been implemented for EV characterization [20,21,22,23,24].

Quantifying the total number of EVs present in a sample has important implications due their dependence on the condition of the mother-cell and the environment [3,7,40,41]. Thus far, the quantification of the total number of EVs has been limited to methods such as NTA and TRPS. In this study, we experimentally showed that QCM biosensors can be used for quantifying the total number of EVs in a sample after the evaporation of the solvent. Compared with NTA and TRPS, this type of biosensor provides a broader range for the EV number (0.03–6 × 10^7^), which it can detect without the need to optimize the sample concentration via dilution. The lowest limit of detection (3 × 10^5^ EVs) is comparable to the NTA limit if the minimum sample volume of 300 µL required for NTA measurements is considered. In addition, the QCM biosensor limits presented in this study are not dependent on the size distribution of the EVs, which is a critical determinant of the measurement accuracy when using NTA and TRPS. Deviations in the results obtained by the biosensor from the NTA results can be attributed to errors related to the manual introduction of the EV sample on the crystal surface as well as the limits of NTA, which originate from a strong decrease in the intensity of the scattered light with the particle diameter, and can lead to an underestimation of the EV concentration [42]. Aggregation [43] and non-specific adsorption during storage [44] can also lead to additional EV losses. However, in this study, the EVs were stored at −20 °C for a short period of time, and the aliquots were only thawed once prior to NTA and QCM characterization, minimizing the contribution of aggregation and adsorption. Deposited mass may also induce dissipative energy losses and lead to an underestimation of the adsorbed film during QCM analysis [45,46]. It was found that in the EV quantity range found to be appropriate for such analysis, the ratio between the change in the dissipation factor and frequency before sample introduction and after its desiccation was below 10^−7^ 1/Hz (Figure S1 in Supplementary Material), so we can conclude that there are minimal contributions leading to underestimation of the adsorbed film that can be caused by dissipative energy losses.

The QCM biosensor takes advantage of the high sensitivity of the mass being deposited on the surface and the linear correlation of the frequency change to the change in the mass per unit area. The EV quantity was shown to be easily determined using the presented method. The volatile electrolyte in 2 mM AA that was applied allowed for EV quantification to be carried out relatively quickly. The minimum time needed to perform a single measurement was primarily determined by the time needed for the complete evaporation of the 2 mM AA solvent. Reducing sample volume and using a more volatile solvent can further decrease the analysis time and only requires appropriate biosensor calibration.

During the evaporation of a droplet on a substrate, a deposit of concentrated particles often tends to appear in the form of a ring-shaped pattern that is commonly referred to as the “coffee ring”. This coffee ring effect was first introduced by Deegan R. D. et al. [47], and this phenomenon has been thoroughly studied both experimentally and theoretically in order to find the determining factors that provide control over the patterns composed of deposited particles [48]. The application of the coffee ring effect has already had an impact on various fields, such as DNA/RNA microarrays, nanoparticle assembly, and biophysical detection [49]. In our study, the EVs in 2 mM AA solvent formed a distinct densely packed ring located at the initial boundary of the sessile drop after desiccation (Figure 3). The gradual frequency decreased to about −400 Hz prior to a rapid increase, and the complete evaporation of the solvent (Figure 2b) was not observed in the results obtained for the control sample only containing 2 mM AA (Figure 2a). In the latter case, an absence of the characteristic decrease in frequency prior to complete evaporation with only a gradual increase in frequency that was significantly longer than in the case of EV sample evaporation was observed. After further inspection, it was found that this difference is primarily due to the pattern formation mechanism during and after solvent evaporation. The EVs in 2 mM AA started with a slow constant contact radius mode (CCR) and ended with a significantly faster slip mode (~30 s) forming a ring-shaped pattern similar to a coffee stain (Figure S2a) [50]. On the other hand, the evaporation of the sessile drop containing only 2 mM AA was more dominated by the constant contact angle (CCA) mode, with the boundary of the drop gradually moving throughout the process. The difference in the speed of the moving boundary could be identified by comparing the slope of the frequency increase in the case of 2 mM AA with and without EVs prior to complete solvent evaporation. When the EV sample reached the transition from the CCR mode to the slip mode that could be identified by the negative peak during solvent evaporation, a thin film with area that was initially occupied by the sessile drop on the quartz crystal formed. As a result, thin film formation by the CCR mode of the EV sample solvent evaporation led to the decrease in frequency and an increase in the resistance and dissipation factor (Figure S1). Imaging the quartz crystal allowed the area of the ring-shaped pattern enriched with EVs on the QCM substrate to be measured in order to find the average mass of the EVs isolated from six different cell lines, and the QCM biosensor determined that the mass was independent from the EV source (Table 3). The agreement between the results obtained by the QCM biosensor and previous reports confirm that EVs form a ring-shaped pattern after sample desiccation, which can be further implemented in future studies related to EV characterization.

## 5. Conclusions

In summary, a novel method for quantifying desiccated EVs using a QCM biosensor was demonstrated. The procedure is simple: a 5 µL volume EV sample is introduced to the center of the quartz crystal surface, and the resonant frequency is monitored before and after the evaporation of the solvent, leaving only a ring-shaped stain that is enriched in EVs. The volatile 2 mM ammonium acetate solvent allows for the analysis time to be reduced due to the rapid evaporation and deposition of EVs on the quartz crystal. The shift in the resonant frequency caused by desiccated EVs obeyed the Sauerbrey equation, with the lower and upper limits being 0.03 and 6 × 10^7^ EVs, respectively. Four samples were used to test the performance of the biosensor and showed agreement with NTA data. The QCM biosensor is adaptive and can be optimized using different volatile solvents or EV sample volumes depending on the end goal. The presented protocol for EV quantification using QCM biosensors is inexpensive, only requires basic training, and can be used routinely in EV studies and for future clinical applications requiring EV characterization.

## Figures and Tables

**Table 1:** NTA characterization of EVs isolated from MCF7, MDA-MB-231, MCF10A, 22Rv1, LNCaP, and PC3 cell culture media showing their mode, mean, and standard deviation of the mean hydrodynamic size (StDev).

	MCF7	MDA-MB-231	MCF10A	22Rv1	LNCaP	PC3
Mode (nm)	117	123	115	106	135	113
Mean (nm)	183	147	205	177	201	220
StDev (nm)	84	74	116	63	141	119

**Table 2:** Results obtained after testing the calibration curve using the obtained relation (Equation (4)) and comparing the results with NTA data. The mean and standard deviation of the mean values represent the number (#) of EVs (three repeats).

	MCF7	MDA-MB-231	LNCaP	PC3
QCM (#) × 10^6^	4.03 ± 0.70	1.14 ± 0.39	19.40 ± 1.05	16.72 ± 1.24
NTA (#) × 10^6^	4.16 ± 0.83	1.05 ± 0.27	21.21 ± 2.04	15.60 ± 0.73

**Table 3:** Mean and standard deviation of the mean (three repeats) mass and diameter of the EVs isolated from all of the cell lines determined by combining the ring area with the Sauerbrey relation and EV quantity in the analyzed samples (5). The density of the EVs used for estimating EV diameter was assumed to be 1.10 g/mL [35,36].

	MCF7	MDA-MB-231	MCF10A	22Rv1	LNCaP	PC3
Ring area (mm^2^)	0.25 ± 0.02	0.15 ± 0.03	0.26 ± 0.04	0.24 ± 0.01	0.29 ± 0.05	0.18 ± 0.02
Mass (fg)	0.16 ± 0.01	0.13 ± 0.03	0.18 ± 0.04	0.16 ± 0.01	0.17 ± 0.04	0.13 ± 0.01
Diameter (nm)	52 ± 1	49 ± 9	54 ± 2	51 ± 1	53 ± 3	48 ± 2

## Data Availability

No new data were created or analyzed in this study. Data sharing is not applicable to this article.

## Supplementary Materials

The following supporting information can be downloaded at: https://www.mdpi.com/article/10.3390/bios12060371/s1, Figure S1: Response in dissipation; Figure S2: Evaporation of a sessile drop in different modes.
