# Supplementary material for: Quantification of Desiccated Extracellular Vesicles by Quartz Crystal Microbalance
Source: Biosensors (Basel). 2022 May 27;12(6):371. doi: 10.3390/bios12060371 (PMC9221410; doi:10.3390/bios12060371)
Supplement: Supplementary file 1 [file biosensors-12-00371-s001.zip › biosensors-1685023-supplementary.pdf]

Electronic supplementary information

Quantification of Desiccated Extracellular Vesicles by Quartz Crystal Microbalance

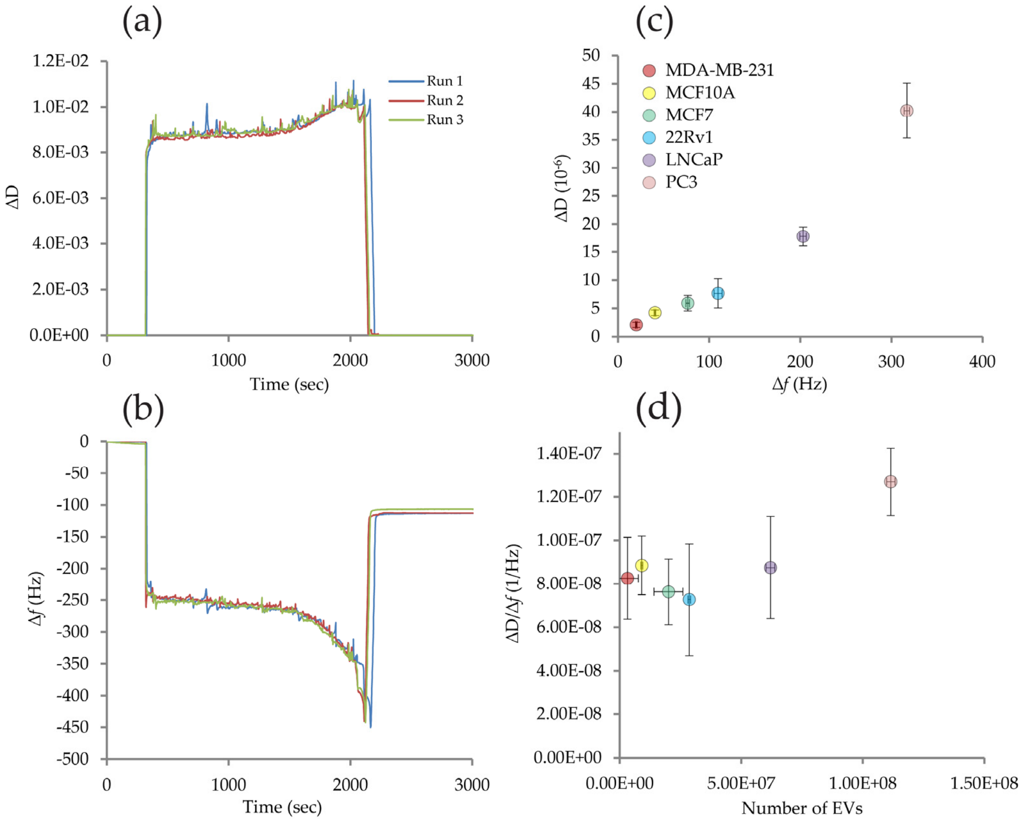

Figure S1. Response in dissipation after a sample of EVs in 2 mM ammonium acetate (AA) is deposited on the quartz crystal. Examples of the dissipation (a) determined by using its relation to the resistance [1,2] and the frequency (b) presented relative to their initial (t = 0 sec) values after QCM analysis of 22Rv1 EVs in 2 mM AA. (c) Dissipation shift obtained by comparing values before EV sample introduction and after its desiccation on the quartz crystal. (d) Ratio of the dissipation and the frequency shift before EV sample introduction and after desiccation relative to the number of EVs present in the sample.

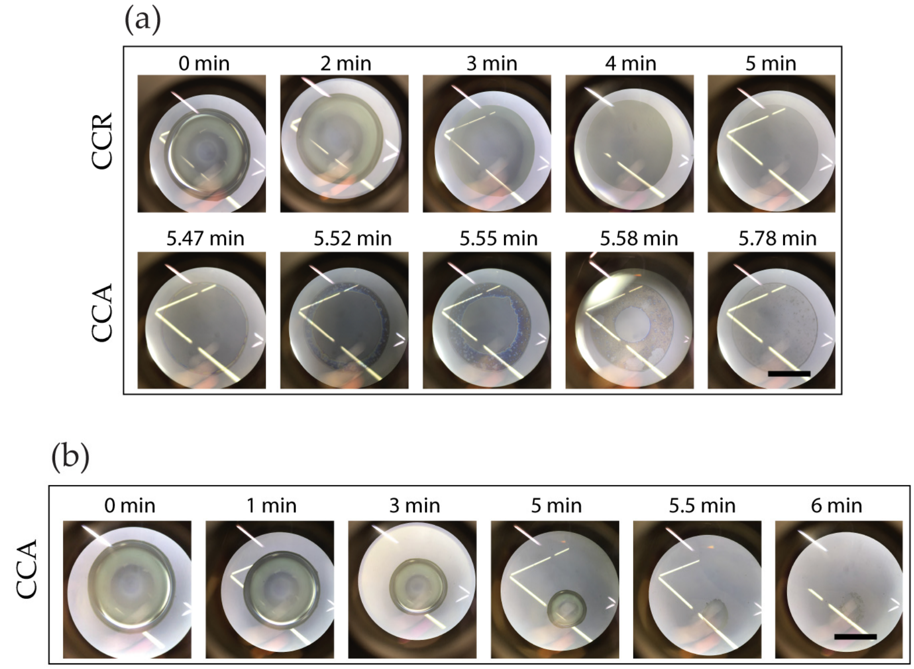

Figure S2. Images obtained by a microscope (Leica DM500, Wetzlar, Germany) showing evaporation of a sessile drop of 2 mM AA with (a) or without (b) EVs with an initial volume of 1 μL deposited on the gold surface. Constant contact radius (CCR) and constant contact angle (CCA) modes were identified after analysis of the obtained images throughout the sessile drop evaporation [3]. The time needed for completion of the CCA mode resulting in desiccated EVs (a) is ~30 sec. Scale bars are 500 μm.

References:

1. Voinova, M. V.; Jonson, M.; Kasemo, B. "Missing mass" effect in biosensor's QCM applications. *Biosens. Bioelectron.* **2002**, *17*.

2. Johannsmann, D.; Reviakine, I.; Richter, R.P. Dissipation in films of adsorbed nanospheres studied by quartz crystal microbalance (QCM). *Anal. Chem.* **2009**, *81*.

3. Parsa, M.; Harmand, S.; Sefiane, K. Mechanisms of pattern formation from dried sessile drops. *Adv. Colloid Interface Sci.* **2018**, *254*.
